# Determinants of apprehension to return to sport after reconstruction of the anterior cruciate ligament: an exploratory observational retrospective study

**DOI:** 10.1186/s13102-022-00433-1

**Published:** 2022-03-15

**Authors:** Alexandre Martini, Anne Ayala, Marc Lechable, François Rannou, Marie-Martine Lefèvre-Colau, Christelle Nguyen

**Affiliations:** 1 AP-HP.Centre-Université Paris Cité, Hôpital Cochin, Rééducation Et Réadaptation de L’Appareil Locomoteur Et Des Pathologies du Rachis, 27, Rue du Faubourg Saint-Jacques, 75014 Paris, France; 2 Centre de Rééducation Fonctionnelle, 94350 Villiers-Sur-Marne, France; 3 Université Paris Cité, Faculté de Santé, UFR de Médecine, 75006 Paris, France; 4 INSERM UMR-S 1124, Toxicité Environnementale, Cibles Thérapeutiques, Signalisation Cellulaire Et Biomarqueurs (T3S), Campus Universitaire des Saints-Pères, 75006 Paris, France; 5 INSERM UMR-S 1153, Centre de Recherche Épidémiologie Et Statistique Paris Sorbonne Cité (CRESS), ECaMO Team, 75004 Paris, France; 6 Institut Fédératif de Recherche Sur Le Handicap, 75013 Paris, France

**Keywords:** Anterior cruciate ligament injury, Return to sport, Apprehension

## Abstract

**Background:**

Only 65% of people return to a level of sport equivalent to that before after anterior cruciate ligament (ACL) surgery. Persisting apprehension may in part explain this observation. We aimed to describe characteristics of people with ACL-Return to Sport after Injury (RSI) scores ≥ 60/100 (low apprehension) at 6 months after injury and to identify variables independently associated with low apprehension at 6 months.

**Methods:**

We conducted a single-center retrospective study. People who had surgery for an ACL rupture and who participated in an outpatient post-operative rehabilitation program were included consecutively. The ACL-RSI questionnaire was self-administered at 6 months after injury. Baseline characteristics of people with ACL-RSI scores ≥ 60/100 and < 60/100 were described. Multiple logistic regression was performed to identify baseline variables associated with low apprehension at 6 months.

**Results:**

We included 37 participants: 13/37 (35.1%) were women and mean age was 27.2 (9.2) years. At 6 months, 21/37 (56.8%) had an ACL-RSI score ≥ 60/100. Participants who had an ACL-RSI score ≥ 60/100 more often received a preoperative rehabilitation (16/21 [76.2%] vs 5/16 [31.2%]), and had less often knee pain (7/21 [33.3%] vs 7/16 [43.7%]) and effusion (5/21 [23.8%] vs 8/16 [50.0%]) at 1 month after surgery, than participants who had an ACL-RSI score < 60/100. In the multivariate analysis, preoperative rehabilitation was associated with low apprehension at 6 months (OR [95% CI] = 0.107 [0.023 to 0.488], *p* = 0.002).

**Conclusions:**

Preoperative rehabilitation was independently associated with low apprehension at 6 months.

*Trial registration*. Not applicable.

**Supplementary Information:**

The online version contains supplementary material available at 10.1186/s13102-022-00433-1.

## Background

Rupture of the anterior cruciate ligament (ACL) is the most frequent knee ligament injury. Its surgical management has increased over the last decade [1, 2]. Approximately 65% of people return to a level of sport equivalent to that before 2 years post-operatively [3]. This rate can reach 83% in elite athletes [4]. The ACL-Return to Sport after Injury (RSI) is a self-administered questionnaire, validated in 2008, specifically designed to quantify the apprehension to return to sport after ACL injury (0 = maximal apprehension and 100 = no apprehension) [5, 6]. An ACL-RSI score ≥ 60/100 at 6 months after injury correlates with a higher rate of returning to sport at 2 years after injury [7]. Therefore, ACL-RSI score at 6 months after injury could be considered as an interim outcome of returning to sport in the long term after ACL injury.

However, determinants of an ACL-RSI score ≥ 60/100 at 6 months after injury have been scarcely explored. In the present study, we aimed to describe characteristics of people with scores ≥ 60/100 and < 60/100 at 6 months after surgery and to identify variables independently associated with an ACL RSI < 60/100 score at 6 months.

## Methods

### Design

We conducted an exploratory observational retrospective single-centered study. Our study is reported in accordance with the Strengthening the Reporting of Observational Studies in Epidemiology (STROBE) statement [8] (Additional file 1).

### Participants

Operated people fulfilling inclusion criteria and participating in the outpatient post-operative rehabilitation program at the rehabilitation center of Villiers-sur-Marne (France), from May 2018 to August 2019, were consecutively included. Inclusion criteria were age greater than 15 years, time elapsed between knee surgery and participation in the rehabilitation program less than 8 days, ACL rupture, either isolated or with meniscal involvement. Non-inclusion criteria were associated posterior cruciate ligament or medial collateral ligament injuries and insufficient proficiency in French. All included patients were operated.

### Interventions

The outpatient program was conducted for 6 weeks at the rehabilitation center by experienced physiotherapists in accordance with the 2008 French *Haute Autorité de Santé* recommendations [1]. Briefly, in the first 3 weeks of the program, patients aim to achieve active locking in extension and 60–90° flexion of the knee. In the next 3 weeks, they aim to achieve 120° of flexion and a painfree knee without effusion. To this end, patients had four 45-min sessions of physiotherapy per week including passive knee mobilisation, closed chain muscular stabilization exercises and proprioception exercises in bipodal then unipodal support, on stable then unstable planes. Water-based exercise therapy was allowed after complete skin healing. At discharge, patients were referred to an outpatient physiotherapist for 2 to 3 sessions a week. The content of the outpatient physiotherapy sessions was left at the discretion of the physiotherapist. Patients received information about the movements to be avoided (e.g. open chain, torsion). Physiotherapists instructed participants to perform home-based exercises including isometric quadriceps contractions and knee flexions during the first two weeks. Returning to sport was not allowed.

### Outcomes

Baseline variables were collected by the same physician (AM) during a medical interview and extracted from medical records. The ACL-RSI questionnaire was self-administered at the 6-month face-to-face follow-up visit with the physiotherapist of the center. If the patient could not attend the 6-month face-to-face visit, he was contacted by the investigator by phone or email and invited to complete and return the ACL-RSI questionnaire by mail or email. The 6-month contact was part of the routine in our center.

### Statistical analyses

Baseline characteristics were described with absolute and relative frequencies (n [%]) for categorical data and means (SD) or median [interquartile range] for continuous data, as appropriate. Because our sample size was small and the risk of biases related to our retrospective design was high, we stuck to descriptive analyses and purposely did not prespecify nor perform any comparative analyses. To identify variables independently associated with an ACL-RSI score < 60/100 at 6 months after surgery, we performed a multiple logistic regression (MYSTAT 12 v12.02.00). We entered 2 baseline variables selected for their clinical relevance: preoperative rehabilitation (i.e. patient self-reporting having received outpatient physiotherapy sessions) and a maximum force deficit in knee extension greater than 20% compared to the contralateral side, when measured isokinetically at a speed of 60°/sec at 6 months postoperatively. Variables were further selected by automatic backward stepwise regression, with *p* = 0.15 to enter in the model and *p* < 0.05 to stay in the final model.

*Ethical consideration.*

Informed consent was obtained for all patients for a record review. Patients were informed that data recorded in medical files may be retrospectively extracted for research purpose. Our Institutional Review Board (Comité des Recherches non CPP, CERAPHP Centre) confirmed on September 11, 2020, that our study did not fall under the Jardé Law, because our data were retrospectively collected. Therefore, an ethical approval was not necessary.

## Results

### Participants

Overall, 37 patients met inclusion criteria and were consecutively included: 13/37 (35%) patients were women, 21/37 (57%) had preoperative rehabilitation, and mean age was 27.2 (9.2) years (7 participants were younger than 18 years). The median (interquartile range) time elapsed between injury and surgery was 11.0 (8.0–22.0) weeks (Table 1).

**Table 1:** Participants’ demographics (N = 37)

Age (years), mean (SD)	27.2 (9.2)
Females, n (%)	13 (35)
Body mass index^a^ (kg/m^2^), mean (SD)	23.8 (2.8)
Time elapsed between injury and surgery^b^ (weeks), median (IQR)	11.0 (8.0–22.0)
Preoperative rehabilitation, n (%)	21 (57)
**Professional status, n (%)**	
Professional athletes	3 (8)
High school students	7 (19)
Employed	26 (70)
Unemployed	1 (3)
**Level of sport**^**c**^**, n (%)**	
Professional	3 (8)
Competition	18 (49)
Leisure	15 (40)
**Type of sport, n (%)**	
Handball	7 (19)
Football	8 (22)
Martial arts	3 (8)
Ski	3 (8)
Racket sport	3 (8)
Other	11 (30)
Traffic accident/work injury	2 (5)
**Surgery, n (%)**	
Isolated anterior cruciate ligament reconstruction	26 (70)
Partial meniscectomy	10 (27)
Meniscal suture	1 (3)
**Surgical technique, n (%)**	
Semitendinosus and gracilis construct	22 (59)
DT4	8 (22)
Mac Intosh	1 (3)
Kenneth Jones	6 (16)
Lemaire	6 (16)

IQR = interquartile range; n = number; SD = standard deviation

^a^n = 36

^b^n = 34

^c^n = 36

### ACL-RSI score

At 6 months after injury, 21/37 (57%) patients had an ACL-RSI score ≥ 60/100 (low apprehension). Patients who had an ACL-RSI score ≥ 60/100 more often received preoperative rehabilitation (16/21 [76%] *vs* 5/16 [31%]), were younger (26.3 [9.3] *vs* 28.2 [9.3] years), had less often knee pain (7/21 [33%] *vs* 7/16 [44%] and less often knee joint effusion (4/21 [19%] *vs* 8/16 [50%]) at 1 month after surgery, than patients who had an ACL-RSI score < 60/100 (high apprehension) at 6 months after injury. They were also more often "competition" sportsmen and women (11 [52%] *vs* 7 [44%]) (Table 2).

**Table 2:** Characteristics of participants with and without apprehension at 6 months

	ACL-RSI ≥ 60 (n = 21)	ACL-RSI < 60 (n = 16)
Age (years), mean (SD)	26.3 (9.3)	28.2 (9.3)
Females, n (%)	5 (24)	8 (50)
Body mass index^a^ (kg/m^2^), mean (SD)	24 (4.1)	23.6 (3.2)
Time elapsed between injury and surgery^b^ (weeks), mean (SD)	15.5 (8.0–17.0)	9.0 (7.5–19.0)
Preoperative rehabilitation, n (%)	16 (76)	5 (31)
**Professional status, n (%)**		
Professional athlete	2 (9)	1 (6)
High school student	4 (19)	3 (19)
Employed	15 (71)	11 (69)
Unemployed	0 (0)	1 (6)
**Level of sport**^**c**^**, n (%)**		
Professional	2 (9)	1 (6)
Competition	11 (52)	7 (44)
Leisure	7 (33)	8 (50)
**Surgery, n (%)**		
Isolated anterior cruciate ligament reconstruction	15 (71)	11 (69)
Partial meniscectomy	5 (24)	4 (25)
Meniscal suture	1 (0)	0 (0)
**Surgical technique, n (%)**		
Semitendinosus and gracilis construct	12 (57)	10 (62)
DT4	6 (29)	2 (12)
Kenneth Jones	2 (9)	4 (25)
Mac Intosh	1 (5)	0 (0)
Lemaire	4 (19)	2 (12)
**Complications at 1 month after surgery**		
Knee pain (yes), n (%)	7 (33)	7 (44)
Knee effusion (yes), n (%)	4 (19)	8 (50)
Amyotrophy (yes), n (%)	15 (71)	10 (62)
Thigh amyotrophy (cm), mean (SD)	2.9 (1)	2.7 (1)
**Quadriceps strength deficit at 6 months after surgery**^**d**^**, mean (SD)**		
Isokinetic test at 90°.s^−1^ (%)	19.6 (11)	33.4 (23)
Isokinetic test at 60°.s^−1^ (%)^d^	19.0 (8)	30.8 (21)

IQR = interquartile range; n = number; SD = standard deviation

^a^n = 36

^b^n = 34

^c^n = 36

^d^n = 33

### Variables associated with ACL-RSI score

In the final multivariate model, preoperative rehabilitation was the variable significantly inversely associated with an ACL-RSI score < 60/100 (high apprehension) at 6 months after surgery (*Odds Ratio* [95% CI] = 0.107 [0.023 to 0.488], *p* = 0.002).

## Discussion

In the present study, we found that baseline characteristics of patients with low apprehension at 6 months after ACL injury differed from those with high apprehension.

Overall, patients with an ACL-RSI score ≥ 60/100 (low apprehension) at 6 months after injury were younger and had less often post-operative knee pain and knee joint effusion than those with an ACL-RSI score < 60/100. Our results were consistent with those of Webster and colleagues, who found a positive effect of younger age and post-operative International Knee Documentation Committee score on the psychological readiness to return to sport [9]. In our study, we observed that nearly half (49%) of the participants were "competitive" athletes, compared to 40% in the study by Sadeqi et al. In addition, 66% practiced a pivot or contact pivot sport compared to 84% in this same study. Finally, concerning the types of surgical procedures, hamstring grafts (ST-G, T4D) represented 81% of the procedures in our study *versus* 88% in Sadeqi et al.

In our study, 21/37 (57%) patients received preoperative rehabilitation. We found that 2/3 (67%) professional sportsmen and women had preoperative rehabilitation, 10/18 (55%) “competition” sportsmen and women, but only 4/16 (25%) “leisure” sportsmen and sportswomen. Several reasons may explain our observation: surgeons and physicians may have had different prescription habits, preoperative rehabilitation protocols could have differed depending on the profile of the referred patient, and surgeons and physicians could have selected patients to preoperative rehabilitation based on their level of sport.

In multivariate analysis, preoperative rehabilitation was associated with ACL-RSI score at 6 months after injury. In the present study, preoperative rehabilitation was not standardized. Rehabilitation programs usually include muscle strengthening (quadriceps/hamstring) and proprioception exercises [10]. These practices stem from the fact that the strength of the quadriceps/hamstring pair in preoperative care is associated with better knee function at 6 months after surgery [11]. There are no recommendations on the optimal duration, intensity, frequency and mode of delivery of preoperative rehabilitation sessions before ACL surgery. A study of non-superiority did not show any benefits in continuing rehabilitation beyond 10 weeks [12].

Our study has limitations. Firstly, one of the main limitations was the use of different surgical techniques with also different surgeons and the practice of very different sports. Secondly, prescription habits of preoperative rehabilitation could have differed between surgeons and one cannot exclude that an operator effect may have influenced the ACL-RSI score at 6 months. Thirdly, outpatient physiotherapy between 6 weeks and 6 months post-operatively was also left at the discretion of the physiotherapist and was not standardized. Lastly, that some participants, contrary to others, could have received home-based exercises and/or suitable physical activity in addition to the supervised sessions, could also have influenced the ACL-RSI score at 6 months. However, the retrospective and exploratory design of our study did not allow us to comprehensively collect these data and to include them in the analyses. Our small sample size did not allow us to perform robust multivariate modelling. Finally, information about performance-related variable such as isokinetic quadriceps strength, history of previous contralateral injury, Tegner score, involved surgeons, and adherence to rehabilitation were not collected, and effect sizes were not calculated, but could have added relevant information to our dataset.

## Conclusions

Patients with low apprehension at 6 months after ACL injury differ from those with persisting apprehension. Our exploratory findings support further research to assess the effectiveness of preoperative rehabilitation to enhance psychological readiness to return to sport after reconstruction of the ACL.

## Supplementary Information

**Additional file 1**. **STROBE** Statement-checklist of items that should be included in reports of observational studies.

## Data Availability

The full original protocol and dataset can be accessed by academic researchers by contacting Prof. Christelle Nguyen (christelle.nguyen2@aphp.fr).

## Abbreviations

ACL: Anterior cruciate ligament
RSI: Return to Sport after Injury
SD: Standard deviation
STROBE: Strengthening the reporting of observational studies in epidemiology (STROBE
